# Association between Exposure to Influenza Vaccination and COVID-19 Diagnosis and Outcomes

**DOI:** 10.3390/vaccines8040675

**Published:** 2020-11-12

**Authors:** Pietro Ragni, Massimiliano Marino, Debora Formisano, Eufemia Bisaccia, Stefania Scaltriti, Emanuela Bedeschi, Roberto Grilli

**Affiliations:** 1 Unit of Clinical Governance, Azienda Unità Sanitaria Locale—IRCCS di Reggio Emilia,42122 Reggio Emilia, Italy; pietro.ragni@ausl.re.it (P.R.); massimiliano.marino@ausl.re.it (M.M.); debora.formisano@ausl.re.it (D.F.); 2 Department of Public Health, Azienda Unità Sanitaria Locale—IRCCS di Reggio Emilia, 42122 Reggio Emilia, Italy; eufemia.bisaccia@ausl.re.it (E.B.); stefania.scaltriti@ausl.re.it (S.S.); emanuela.bedeschi@ausl.re.it (E.B.)

**Keywords:** influenza vaccine, SARS-Cov-2, COVID-19, case-negative study, retrospective cohort study

## Abstract

We explored whether influenza vaccination (IV) affects susceptibility to SARS-CoV-2 infection and clinical outcomes in COVID-19 patients in 17,608 residents of the Italian province of Reggio Emilia undergoing a SARS-CoV-2 test. Exposure to IV was ascertained and the strength of the association with SARS-CoV-2 positivity expressed with odds ratios (OR). Rates of hospitalisations and death in those found positive were assessed and hazard ratios (HR) were estimated. The prevalence of IV was 34.3% in the 4885 SARS-CoV-2 positive and 29.5% in the 12,723 negative subjects, but the adjusted OR indicated that vaccinated individuals had a lower probability of testing positive (OR = 0.89; 95% CI 0.80–0.99). Among the 4885 positive individuals, 1676 had received IV. After adjusting for confounding factors, there was no association between IV and hospitalisation (1.00; 95% CI 0.84–1.29) or death (HR = 1.14; 95% CI 0.95–1.37). However, for patients age ≥65 vaccinated close to the SARS-CoV-2 outbreak, HRs were 0.66 (95% CI: 0.44–0.98) and 0.70 (95% CI 0.50–1.00), for hospitalisation and death, respectively. In this study, IV was associated with a lower probability of COVID-19 diagnosis. In COVID-19 patients, overall, IV did not affect outcomes, although a protective effect was observed for the elderly receiving IV almost in parallel with the SARS-CoV-2 outbreak. These findings provide reassurance in planning IV campaigns and underscore the need for exploring further their impact on COVID-19.

## 1. Introduction

In the context of the COVID-19 pandemic, influenza vaccination campaigns are likely to be more important than ever in the near future, not least to protect health services from having to deal with the burden of an influenza epidemic while facing the challenges of COVID-19. International health agencies have authoritatively pointed out the necessity of making every possible effort to sustain already established immunisation programmes [1,2]. Further, achieving high immunisation rates against influenza is specifically needed in order to preserve both the stability of health care systems and the health of the population [3,4].

So far, the relationship between influenza vaccine administration and susceptibility to SARS-CoV-2 has been little explored. Only a recent Italian study—a survey based on a self-administered questionnaire—documented that participants aged <65 years old who had reported receiving an influenza vaccination were less likely to test positive in the SARS-Cov-2 test than those not vaccinated [5].

As for outcomes in COVID-19 patients, the association with the influenza vaccine has been explored so far mainly in correlational studies [6,7,8,9], and more recently in a large cohort of COVID-19 patients, in which exposure to the influenza vaccine was found to be associated with lower severity and mortality [10].

In the province of Reggio Emilia (northern Italy, population 539,652), an influenza vaccination campaign is launched yearly from October to March that especially targets those over the age of 65.

In this report, we assessed whether administration of the influenza vaccine during the last campaign had any relationship with susceptibility to SARS-CoV-2 infection (i.e., the likelihood of being diagnosed with COVID-19) and whether rates of outcomes in all the COVID-19 patients diagnosed differed according to their exposure to the influenza vaccination.

## 2. Materials and Methods

### 2.1. Study Design

We conducted a test-negative study [11], with all those found positive for the SARS-CoV-2 RNA by polymerase chain reaction on nasopharyngeal/throat swab as cases, and those negative as controls. The two groups were compared for their previous exposure to the influenza vaccination.

Then, cases represented the cohort of all patients diagnosed COVID-19 who were followed up over time, with rates of hospitalisations and death assessed and compared between those who had been exposed and unexposed to the influenza vaccination. In/out-of-hospital deaths due to any cause and hospitalisation were the primary end points of this retrospective cohort study. Outcome measures were, therefore, time from date of test execution to these events. For this report, follow-up was closed on 18 June 2020, with a median length of 30 days (range 1 to 106).

### 2.2. Study Population and Data Sources

For this study we considered all the 17,790 residents who underwent the nasal and oropharyngeal swab test for SARS-Cov-2 in the province of Reggio Emilia between 15 February and 22 May 2020. COVID-19 was diagnosed in 27.5% (4895) of this population.

Data on all the residents undergoing the swab test were retrieved from the COVID-19 surveillance database, developed in the province at the inception of the pandemic. This registry contains information about the date of test execution, diagnosis, hospitalisations, emergency room and intensive care unit (ICU) admissions, and deaths of all the patients testing positive for SARS-CoV-2 RNA by polymerase chain reaction on nasopharyngeal/throat swabs.

This SARS-CoV-2 database was linked with the administrative databases available to the Local Health Authority, including, for each resident in the province, demographic information, hospital discharge data (coded according to the International Classification of Diseases-9-CM (ICD-9-CM)) of diagnosis and procedures, admission and discharge dates, vital status at discharge, and outpatient pharmacy data at the individual prescription level. Data were anonymized, and record linkage procedures were performed according to the unique identification number which was assigned to each resident. We searched for information on previous hospitalisations (in the preceding 10 years), as registered in these local administrative databases, in order to estimate individual patients’ degree of comorbidity (if any), according to the (not age-adjusted) Charlson index [12].

### 2.3. Exposure

Previous exposure to influenza vaccine was ascertained through record linkage with the provincial registry of vaccinations, including all the 96,940 residents to whom the influenza vaccine was administered during the last (October 2019–March 2020) vaccination campaign. As this linkage was not possible for 182 (1.0%) tested residents, 17,608 were included in the analysis.

Two inactivated vaccines were used in the 2019–2020 influenza immunisation campaign: a split quadrivalent and an adjuvanted subunit trivalent vaccine. The first includes two type A (H1N1 e H3N2) and type B (Colorado/06/2017 and Phuket 3073/2013) strains. The trivalent vaccine adjuvanted with MF59C.1 includes two type A strains (H1N1 and H3N3) and the type B/Colorado/06/2017 strain, and its use is targeted to the elderly and to those with primary or secondary immunodeficiency, according to the recommendations provided by the Italian Ministry of Health [13].

### 2.4. Statistical Analysis on Susceptibility to SARS-CoV-2

The comparison between cases and controls was adjusted through a multivariate logistic regression model, with positivity for the SARS-CoV-2 swab test as the dependent variable. Sex, age, and Charlson index score were the covariates of interest. Age was categorised in 5 classes (≤50, 51–60, 61–70, 71–80, >80), and the Charlson index in 4 (0, 1, 2, and ≥3). Categorical variables were expressed as percentages, and the chi-square test was used to compare exposed vs. unexposed to the influenza vaccine.

In addition, we included in the multivariate logistic model, the time during the pandemic in which the swab test was executed. This variable was intended to take into account both the effect of the accessibility to the SASR-CoV-2 swab test and the prevalence of the disease. Both these factors varied over time during the pandemic. Criteria for test accessibility were more selective (i.e., testing only symptomatic cases and their close contacts) in the early weeks, becoming gradually more extensive later. Disease prevalence was higher in the first four weeks, then gradually decreased under the effect of the policies of social distancing and the general lockdown implemented at the national level.

We also assessed whether—in the source population from which cases and controls were drawn (vaccinated and unvaccinated)—there was comparable accessibility to the SARS-CoV-2 swab test over the entire pandemic period. The resident population was divided into subgroups according to age, sex, and influenza vaccine administration and for each of the seven time intervals, relying on the number of individuals tested; an odds ratio (OR) representing the likelihood for vaccinated vs. unvaccinated subjects of being tested was estimated. Time interval-specific ORs were then pooled to estimate an overall measure of the association between exposure to influenza vaccination and accessibility to the swab test at the population level. Overall, we found that vaccinated subjects had only a slightly (although statistically significant) higher probability of being tested for SASR-Cov-2 (OR = 1.04; 95% CI 1.00–1.08), without variations across the different temporal phases of the pandemic but with significant variations across age groups. In particular, vaccinated subjects age ≤50 had a much higher probability of receiving the swab test, while those vaccinated age >80 had a much lower probability (see Section S1 and S2 in Supplementary Material).

### 2.5. Statistical Analysis on COVID-19 Patient Outcomes

Crude (i.e., unadjusted) rates of relevant clinical events (admission to emergency room, hospitalisation, admission to ICU, death) in both groups were compared, and a crude hazard ratio (HR) was estimated to express the strength of the association (if any) between exposure to influenza vaccination and the outcomes of interest. The HR was then adjusted through multivariate regression analyses on time to event, using the Cox’s proportional hazard model [14]. Adjusted hazard ratios (HRadj) with 95% confidence intervals (95% CI) were estimated for death and hospitalisation. Included as covariates in the model, were exposure to influenza vaccination, age, and comorbidity.

All the analyses were conducted both on the whole study population and on the subgroup of those aged ≥65, i.e., those to whom vaccination campaigns are especially targeted (in this case age was treated as a continuous variable).

Secondary exploratory analyses considered the effect of different types of influenza vaccine (trivalent vs. tetravalent) and timing of vaccine administration, distinguishing between those who received immunisation early on in the last immunisation campaign (October–November 2019) vs. those to whom the vaccine was administered later (from December 2019 to March 2020), that is, much closer to the SARS-CoV-2 outbreak.

### 2.6. Ethics

The study was approved by the Area Vasta Emilia Nord Ethical Committee on 07/04/2020 n° 2020/0045199; and on 09/06/2020 n° 2020/0069622.

## 3. Results

The general characteristics of the 17,608 residents who underwent the SARS-CoV-2 test from SARS-CoV-2 outbreak to 22 May 2020 in the province of Reggio Emilia are reported in Table 1, according to swab test results. A further description of the characteristics of those undergoing the swab test over the different phases of the pandemic period is provided in Section S2 of the Supplementary Material.

Positive and negative subjects did not differ in terms of sex, but those who were negative were relatively younger. While comorbidities were not ascertainable for 1472 (11.6%) and 511 (10.4%) in the negative and positive group, respectively, the proportion of those without relevant comorbidities was relatively higher for those testing negative (76% vs. 71%).

Previous exposure to influenza vaccine administration was more frequently detected among those who tested positive (34.2% vs. 29.5%). The influenza vaccine (the tetravalent in 60.2% of the cases) was administered between October and November 2019 in 85.5% of cases.

Prevalence of SARS-CoV-2 positivity was 26.3% in unvaccinated and 30.9% in vaccinated subjects, whose probability of being found positive when tested was therefore higher by 26% (OR = 1.26; 95% CI 1.17–1.34) (see Table 2). After adjusting for age, sex, Charlson index, and time when the swab test was performed, the OR indicated that exposure to influenza vaccination was associated with a (marginally) statistically significant reduction in the likelihood of testing positive on the swab test (OR = 0.89; 95% CI 0.80–0.99); the same held true in the subgroup of patients of age ≥65 (Table 2).

As for the type of vaccine and the timing of its administration, a lower likelihood of being positive was again associated with the tetravalent vaccine and to an early (i.e., in October–November 2019) administration (Table 2).

### Outcomes in COVID-19 Patients

The characteristics of the 4885 COVID-19 patients are reported in Table 3.

As expected, the 1676 patients who received the influenza vaccine were older and had more comorbidities, as represented by the Charlson index. The latter was missing for 369 patients (7.5%), for whom the record linkage procedure required for the Charlson index estimation was unfeasible; 315 of them were unvaccinated. Hypertension, cancer and diabetes were the more frequently observed comorbidities in COVID-19 patients (in 18%, 11% and 10% of the cases, respectively), while other conditions (including chronic obstructive pulmonary disease, obesity, heart and renal failure) were present in less than 10% of the patients. All comorbidities—except for cancer—were consistently more common among vaccinated individuals.

As for type of influenza vaccine, trivalent and tetravalent were almost equally represented (692 and 898 patients, respectively), although these two groups differed, as expected, in terms of patient characteristics (Table 4).

The trivalent vaccine was in fact largely used in the elderly, while the group receiving the tetravalent vaccine had a relatively higher proportion of males (48.7% vs. 36.1%) and of patients without any comorbidity (58.7% vs. 34.5%). Overall, only a minority of patients (232, 13.8%) had the vaccine administered later during the last immunization campaign.

Overall, 860 (51.1%) and 1676 (52.1%) patients, respectively, for vaccinated and unvaccinated, visited an emergency room at the onset of their disease, with immediate hospital admission for 370 (22.0%) vaccinated and 364 (11.3%) unvaccinated patients. The crude risk of hospitalisation over time for the remaining patients (see Figure S1 in Section S3 of the Supplementary Material) was consistently higher for those who were vaccinated. The same held true for the crude cumulative risk of death (Figure S2, in Section S3 of the Supplementary Material).

The strength of the association of exposure to influenza vaccination and death is reported in Table 5, where univariate and multivariate analyses are outlined.

The crude HRs indicated an increased risk of death for those exposed to the influenza vaccination, but the adjusted HRs comparing exposed vs. unexposed individuals indicated a lack of association, both in the overall sample (HRadj = 1.14; 95% CI 0.95–1.31) and in those age ≥65 years (HRadj = 1.04; 95% CI 0.86–1.25). Secondary multivariate analyses showed a statistically significant higher risk for those who received the trivalent vaccine and a 30% reduction in the risk of death (HR = 0.70; 95% 0.50–1.00) for those elderly patients receiving the vaccine later in the immunization campaign.

The same pattern of results emerged for hospital admissions (Table 6).

There was no association between vaccination exposure and hospital admissions in the main multivariate analyses: HRadj were 1.00 (95% CI 0.84–1.19) and 0.92 (95% CI 0.75–1.12) for the whole sample and for the subgroup over age 65, respectively. In secondary analyses, for late receivers vs. unexposed individuals there was a risk reduction of about 30%, both overall and for those over the age of 65, with HRs 0.72 (95% CI 0.50–1.05) and 0.66 (95% CI 0.44–0.98), respectively. No benefit associated with influenza vaccination was visible for early receivers.

## 4. Discussion

According to our findings, exposure to influenza vaccination was associated with a lower susceptibility to SARS-CoV-2 infection. We also found no evidence that influenza vaccination affected the outcomes of COVID-19 patients except for a reduction in the risk of hospital admission and death, seen in those aged ≥65 to whom the vaccine was administered almost in parallel with the SARS-CoV-2 outbreak. Although individuals exposed to influenza vaccination were more frequently positive in the SARS-CoV-2 swab test, and COVID-19 patients had higher rates of hospitalisation and death compared to those unexposed, this was due to differences between the two groups. The elderly (those more frequently vaccinated) were more likely to be found positive, being more frequently tested in the earlier phase of the pandemic, when the likelihood of being found positive was higher. Vaccinated COVID-19 patients were much older and had co-existing diseases, both of which are known to be associated with poor outcomes in COVID-19- patients [15,16,17,18,19].

The protective effect of previous exposure to the influenza vaccination on SARS-CoV-2 susceptibility is consonant with recent findings [5], and if furtherly confirmed, might have relevant implications from a public health perspective. Surely this also calls for research aimed at exploring how SARS-CoV-2 and influenza virus interact at the cellular and molecular level. Of course, there may well be alternative explanations to our findings, calling into question the role of factors we did not take into account. Those who decided to have the influenza vaccine administered could have attitudes that are different from those who were not vaccinated; the former may be less prone to adopting behaviours that put their health at risk and, therefore, more likely to comply with the basic behavioural rule of social distancing. Our finding could have also been biased by a different rate of access to the swab test between those who were vaccinated and not vaccinated. While at the provincial population level, vaccinated residents overall had a relatively higher probability of being tested for SARS-CoV-2 (and thus of being found positive), there was substantial variations across age groups, and the very elderly (i.e., >80) vaccinated had a lower probability of undergoing the swab test.

Our secondary analyses were affected by the size of the sample. Concerning susceptibility to SARS-CoV-2, a statistically significant risk reduction was seen for the tetravalent vaccine (used in 60% of our study population) but not for the trivalent, and for early (85% of the sample), but not for late receivers. Nevertheless, the direction of the effect was similar.

As for COVID-19 outcomes, in secondary analyses we found an increased risk in death associated with the trivalent vaccine when compared to patients who were unexposed, which is at odds with the other results. However, given that the trivalent vaccine is more frequently used in the elderly and in those with immunodeficiency [13], the only reasonable explanation for this finding is the persistence of residual confounding factors that our adjustment procedures could not remove.

Hypotheses that influenza immunisation might even have a positive effect on contracting the SARS-CoV-2 infection and on the prognosis of COVID-19 patients are emerging [20]. Further, it has been suggested that influenza might worsen the outcome of COVID-19 patients [21], favouring SARS-CoV-2 penetration in lung tissue through upregulation of the ACE2 receptor [22], a finding that, if confirmed, could lead to hypothesising a positive effect of the influenza vaccine. While studies exploring whether, and how, they interact at the biological and molecular level are ongoing, co-infection by both viruses has been reported in series of cases [23,24,25,26,27,28], but the actual frequency of this phenomenon has yet to be assessed, as well as its clinical implications [29].

Indeed, in our study, evidence of a positive effect associated with previous exposure to the influenza vaccination was found only when comparing exposed vs. unexposed patients according to timing of vaccine administration, with a trend towards a reduction in the risk of death and hospitalisation for those who received the vaccine later in the immunisation campaign which was particularly evident among those >50. This finding is based on secondary analyses on the small number of patients who received the vaccine later (*N* = 232), and it should not be over emphasised. However, it is in line with observations from a larger cohort of COVID-19 patients [10], and it could suggest that if there is any beneficial effect of being exposed to the influenza vaccination, that it does not last long, thus calling into question the role of innate immunity [30].

Overall, at worst, influenza vaccine administration has no influence on COVID-19 diagnosis and outcome; while at best, it might even have some protective effect. In this context, even the worst-case scenario of a lack of association would be a positive message to be conveyed to the general public. Vaccination campaigns are frequently surrounded by fake news and unjustified claims, which are rapidly disseminated through social media. The prevalence of misinformation on, and the degree of hesitancy about, vaccines is high among the general public [31], possibly affecting even the acceptance of a future vaccination campaign against COVID-19 itself [32]. Providing evidence-based information that immunisation is not harmful is an essential component of health policies aimed at gaining public trust, and therefore of bolstering adherence to the immunisation campaigns to be launched in the near future, as misperception of risks has been shown to be one of the reasons for hesitancy [33].

Our study might have several limitations. Accuracy of exposure ascertainment is a reasonable concern, and in our study it was based on record linkage procedures between the COVID-19 database and the provincial registry in which all the influenza vaccine administrations are recorded; this procedure was not feasible only in 172 cases. Nevertheless, when conducting this study, we did not have any information on possible exposure to vaccines other than influenza. More importantly, in our case vs. control comparison we did not have any information on whether individuals who underwent the SARS-CoV-2 swab test were tested because they were symptomatic or because they were contacts of already diagnosed cases.

Although we were able to consider the whole provincial population of residents who had access to the SARS-CoV-2 swab test and all the patients who were diagnosed with COVID-19, the relatively small sample size prevented more robust and deeper subgroup analyses, and might have missed a possible protective effect of vaccination on clinical outcomes.

Additionally, we relied to a large extent, on information drawn from administrative databases, thus lacking detailed information on the clinical characteristics of individual patients and on the degree of severity of their condition. We were therefore not able to adjust the comparison between exposed and unexposed in our cohort of patients in terms of COVID-19 severity.

Lastly, 1778 (15%) individuals among those undergoing the SARS-CoV-2 swab test and 380 (7.7%) of the cohort of COVID-19 patients had to be excluded from the multivariate analyses because of missing information about their degree of comorbidity. Most were in the unexposed group, and 65% were age ≤50. Had they been included in our analyses, our findings would have been completely different if most of them had relevant comorbidities (i.e., Charlson index ≥2), albeit a highly unlikely case given the age distribution.

## 5. Conclusions

According to our findings, exposure to influenza vaccination was associated with a relatively lower probability of testing positive in the SARS-CoV-2 swab test and, in COVID-19 patients, did not affect outcomes (i.e., death and hospital admission). Moreover, a protective effect was observed in the small subgroup of COVID-19 patients who received the vaccine almost in parallel with the SARS-CoV-2 outbreak. Overall, these findings provide reassurance in planning future influenza vaccination campaigns and underscore the need for clinical and epidemiological investigations into how influenza vaccination impacts on COVID-19.

## Figures and Tables

**Table 1:** Characteristics of residents in the Italian province of Reggio Emilia undergoing SARS-CoV-2 swab test from pandemic inception to 22 May 2020, according to swab test result.

SARS-CoV-2 Test		Negative (*N* = 12,723)		Positive (*N* = 4885)		*p*-Value
		N	%	N	%	
**Sex**	male	5744	45.1	2154	44.1	
	female	6979	54.9	2731	55.9	0.201
**Age**	≤50	5747	45.2	1591	32.6	
	51–60	2012	15.8	891	18.2	
	61–70	1504	11.8	636	13.0	
	71–80	1481	11.6	633	13.0	
	>80	1979	15.6	1134	23.2	<0.001
**Charlson Index**	0	8212	64.5	3131	64.1	
	1	939	7.4	437	8.9	
	2	941	7.4	376	7.7	
	≥3	1159	9.1	440	9.0	
	unknown	1472	11.6	501	10.3	0.004
**Influenza vaccination**	Not vaccinated	8972	70.5	3209	65.7	
	October–November 2019	3205	25.2	1444	29.8	
	December 2019–March 2020	546	4.3	232	4.7	<0.001
**Type of vaccine**	Trivalent	1159	9.1	688	14.1	
	Tetravalent	2372	18.6	899	18.4	
	unknown	220	1.7	89	1.8	<0.001

**Table 2:** Swab test, according to previous exposure to influenza vaccine (IV). Secondary analyses are in gray background

		Univariate		Multivariate
	**OR**	**95% CI**	**OR** ^a^	**95% CI**
No exposure	1		1	
Exposure to IV	1.26	1.17–1.34	0.89	0.80–0.99
**By type of vaccine**				
No exposure	1		1	
Trivalent	1.66	1.50–1.84	1.12	0.96–1.31
Tetravalent	1.06	0.97–1.15	0.82	0.73–0.92
**By timing of exposure**				
No exposure	1		1	
Early receivers ^b^	1.26	1.17–1.36	0.89	0.80–0.99
Late receivers ^c^	1.18	1.01–1.39	0.92	0.75–1.13
**In age ≥65 only**				
No exposure	1		1	
Exposure to IV	0.91	0.82–1.02	0.87	0.75–1.00
**By type of IV**				
No exposure	1		1	
Trivalent	1.15	1.01–1.30	1.04	0.87–1.24
Tetravalent	0.75	0.65–0.85	0.77	0.65–0.91
**By timing of exposure**				
No exposure	1		1	
Early receivers ^b^	0.90	0.80–1.00	0.86	0.75–1.00
Late receivers ^c^	1.01	0.83–1.24	0.93	0.71–1.16

^a^: odds ratio adjusted for age, comorbidity, and time of execution of the swab test over the pandemic period; ^b^: early receivers are patients to whom influenza vaccine was administered in the period October–November 2019; ^c^: late receivers are patients to whom influenza vaccine was administered in the period December 2019–March 202.

**Table 3:** COVID-19 patients diagnosed in the province of Reggio Emilia (Italy) since pandemic inception to 22 May 2020, according to previous exposure to influenza vaccination.

Characteristics	No Exposure to Influenza Vaccine	Exposure to Influenza Vaccine	*p*-Value
	**N (%)**	**N (%)**	
**Sex**			
Female	1781 (55.5)	950 (56.7)	
Male	1428 (44.5)	726 (43.3)	0.45
**Age**			
≤50	1442 (44.9)	149 (8.9)	
51–60	745 (23.2)	146 (8.7)	
61–70	403 (12.6)	233 (13.9)	
71–80	265 (8.3)	368 (22.0)	
>80	354 (11.0)	780 (46.5)	<0.001
**Comorbidities (Charlson Index)**			
0	2379 (74.2)	812 (48.4)	
1	206 (6.4)	270 (16.1)	
2	159 (4.9)	223 (13.3)	
≥3	147 (4.5)	320 (19.1)	
Unknown	315 (9.9)	54 (3.2)	<0.001
**Type of vaccine**			
Trivalent		688 (41.0)	
Tetravalent		899 (53.6)	

**Table 4:** COVID-19 patients who received influenza immunisation, according to type of vaccine administered.

Characteristics	Trivalent N 692	Tetravalent N 898	Not Ascertainable	*p*-Value
	*N* (%)	*N* (%)	*N* (%)	
**Sex**				
Female	442 (63.9)	461 (51.3)	47 (54.6)	
Male	250 (36.1)	437 (48.7)	39 (45.3)	<0.001
**Age**				
≤50	2 (<1)	139 (15.5)	8 (9.3)	
51–60	9 (1.3)	134 (14.9)	3 (3.4)	
61–70	27 (3.9)	190 (21.2)	17 (19.7)	
71–80	136 (19.7)	212 (23.6)	19 (22.1)	
>80	518 (74.9)	223 (24.8)	39 (45.3)	<0.001
**Comorbidities (Charlson Index)**				
0	239 (34.5)	527 (58.7)	43 (50.0)	
1	156 (22.5)	102 (11.4)	12 (13.9)	
2	106 (15.3)	102 (11.4)	15 (17.4)	
≥3	173 (25.0)	132 (14.7)	15 (17.4)	
**Unknown**	18 (2.6)	35 (3.9)	1 (1.1)	<0.001
**Timing of vaccine**				
Early receivers ^a^	610 (88.2)	760 (84.6)	74 (86.0)	
Late receivers ^b^	82 (11.8)	138 (15.4)	12 (14.0)	0.131

^a^: early receivers are patients to whom influenza vaccine was administered in the period October–November 2019; ^b^: late receivers are patients to whom influenza vaccine was administered in the period December 2019–March 2020.

**Table 5:** Results of univariate and multivariate analyses on time from diagnosis to death, according to previous exposure to influenza vaccine (IV), in whole cohort of covid-19 patients, and in the age group > 65 years Secondary analyses in gray background.

		Univariate		Multivariate
In the Whole Cohort	HR	95% CI	HRadj ^a^	5% CI
No exposure	1		1	
Exposure to IV	3.81	3.21–4.51	1.14	0.95–1.37
**By type of vaccine**				
No exposure	1		1	
Trivalent	5.86	4.86–7.08	1.27	1.04–1.56
Tetravalent	2.43	1.95–3.02	1.04	0.83–1.30
**By timing of exposure**				
No exposure	1		1	
Early receivers ^b^	3.97	3.34–4.73	1.18	0.97–1.43
Late receivers ^c^	2.81	2.01–3.93	0.80	0.57–1.14
**In age ≥ 65 only**				
No exposure	1		1	
Exposure to IV	1.32	1.11–1.58	1.04	0.86–1.25
**By type of vaccine**				
No exposure	1		1	
Trivalent	1.64	1.35–1.99	1.11	0.90–1.37
Tetravalent	1.01	0.81–1.28	0.99	0.78–1.25
**By timing of exposure**				
No exposure	1		1	
Early receivers	1.39	1.16–1.66	1.09	0.91–1.32
Late receivers	0.94	0.67–1.33	0.70	0.50–1.00

a: adjusted for age, sex, comorbidity; ^b^: early receivers are patients to whom influenza vaccine was administered in the period October–November 2019; ^c^: late receivers are patients to whom influenza vaccine was administered in the period December 2019–March 202. HR: hazard ratios.

**Table 6:** Results of univariate and multivariate analyses on time from diagnosis to hospital admission, according to previous exposure to influenza vaccination (IV), in the whole cohort of covid-19 patients and in the age > 65 years subgroup. Secondary analyses in gray background.

	Univariate		Multivariate	
In the Whole Cohort	HR	95% CI	HRadj ^a^	95% CI
No exposure	1		1	
Exposure to IV	1.78	1.53 to 2.07	1.00	0.84 to 1.19
**By type of vaccine**				
No exposure	1		1	
Trivalent	1.95	1.60 to 2.37	0.93	0.78 to 1.25
Tetravalent	1.65	1.37 to 1.99	1.02	0.84 to 1.26
**By timing of exposure**				
No exposure	1		1	
Early receivers ^b^	1.86	1.59 to 2.18	1.06	0.88 to 1.28
Late receivers ^c^	1.34	0.94 to 1.91	0.72	0.50 to 1.05
**In age ≥ 65 only**				
No exposure	1		1	
Exposure to IV	0.86	0.72 to 1.05	0.92	0.75 to 1.12
**By type of vaccine**				
No exposure	1		1	
Trivalent	0.83	0.66 to 1.03	0.98	0.77 to 1.25
Tetravalent	0.92	0.73 to 1.17	0.90	0.70 to 1.14
**By timing of exposure**				
No exposure	1		1	
Early receivers	0.91	0.75 to 1.11	0.98	0.80 to 1.21
Late receivers	0.63	0.43 to 0.93	0.66	0.44 to 0.98

^a^: adjusted for age, sex, comorbidity; ^b^: early receivers are patients to whom influenza vaccine was administered in the period October–November 2019; ^c^: late receivers are patients to whom influenza vaccine was administered in the period December 2019–March 2020.

## Supplementary Materials

Supplementary material to the paper is available online at https://www.mdpi.com/2076-393X/8/4/675/s1,Section S1: Probability of undergoing SARS-CoV-2 swab test for vaccinated vs. unvaccinated subjects in the population of the Italian province of Reggio Emilia, Section S2: Characteristics of subjects undergoing SARS-CoV-2 swab test from pandemic inception to 22 May 2020 in the Italian province of Reggio Emilia; Section S3: Time from diagnosis to hospital admission and death for COVID-19 patients exposed and unexposed to influenza vaccination. Figure S1: Time from diagnosis to hospital admission (Figure S1) and to death (Figure S2) for covid-19 patients exposed and unexposed to influenza vaccination.
