# Supplementary material for: Association between Exposure to Influenza Vaccination and COVID-19 Diagnosis and Outcomes
Source: Vaccines (Basel). 2020 Nov 12;8(4):675. doi: 10.3390/vaccines8040675 (PMC7711765; doi:10.3390/vaccines8040675)
Supplement: Supplementary file 1 [file vaccines-08-00675-s001.pdf]

Supplementary Material

Association between Exposure to Influenza Vaccination and COVID-19 Diagnosis and Outcomes

Pietro Ragni [1], Massimiliano Marino [1], Debora Formisano [1], Eufemia Bisaccia [2], Stefania Scaltriti [2], Emanuela Bedeschi [2] and Roberto Grilli [1,*]

[1] Unit of Clinical Governance, Azienda Unità Sanitaria Locale—IRCCS di Reggio Emilia,42122 Reggio Emilia, Italy; pietro.ragni@ausl.re.it (P.R.); massimiliano.marino@ausl.re.it (M.M.); debora.formisano@ausl.re.it (D.F.)

[2] Department of Public Health, Azienda Unità Sanitaria Locale—IRCCS di Reggio Emilia, 42122 Reggio Emilia, Italy; eufemia.bisaccia@ausl.re.it (E.B.); stefania.scaltriti@ausl.re.it (S.S.); emanuela.bedeschi@ausl.re.it (E.B.)

* Correspondence: grillir@ausl.re.it

Index

Section S1

Probability of undergoing SARS-CoV-2 swab test for vaccinated vs unvaccinated subjects in the population of the Italian province of Reggio Emilia –

Males **pag 3**

Females **pag 5**

Section S2

Characteristics of subjects undergoing SARS-CoV-2 swab test from pandemic inception to May 22, 2020 in the Italian province of Reggio Emilia – **pag 7**

Section S3

Time from diagnosis to hospital admission and death for covid-19 patients exposed and unexposed to influenza vaccination – **pag 8**

Section S1. Males' probability of undergoing SARS-CoV-2 swab test for vaccinated vs unvaccinated, by age group and time from pandemic onset to May 22, 2020

	N Vaccinated Tested	*N* Unvaccinated Tested	*N* Total Vaccinated	N Total Unvaccinated	OR	L95%CI	U95%CI
**Male <50**							
From pandemic inception to March 16	11	149	3932	152,321	2.86	1.4	5.27
March 17–March 22	36	252	3896	152,069	5.58	3.81	7.94
March 28–April 15	39	270	3857	151,799	5.68	3.95	7.99
April 6–April 16	52	541	3805	151,258	3.82	2.81	5.1
April 17–April 27	48	638	3757	150,620	3.02	2.2	4.06
April 28–May 7	22	487	3735	150,133	1.82	1.13	2.79
May 8–May 22	53	599	3682	149,534	3.59	2.66	4.77
**51–60**							
From pandemic inception to March 16	25	96	4412	19,069	1.13	0.69	1.77
March 17–March 22	29	176	4383	18,893	0.71	0.46	1.06
March 28–April 15	28	116	4355	18,777	1.04	0.66	1.59
April 6 –April 16	35	151	4320	18,626	1	0.67	1.45
April 17–April 27	37	203	4283	18,423	0.78	0.54	1.12
April 28–May 7	28	144	4255	18,279	0.84	0.54	1.26
May 8–May 22	45	189	4210	18,090	1.02	0.72	1.43
**61–70**							
From pandemic inception to March 16	42	69	9406	19,096	1.24	0.82	1.84
March 17–March 22	41	120	9365	18,976	0.69	0.47	1
March 28–April 15	54	59	9311	18,917	1.86	1.26	2.74
April 6–April 16	63	95	9248	18,822	1.35	0.96	1.88
April 17–April 27	71	109	9177	18,713	1.33	0.97	1.81

April 28–May 7	61	91	9116	18,622	1.37	0.97	1.92
May 8–May 22	91	122	9025	18,500	1.53	1.15	2.02
**71-70**							
From pandemic inception to March 16	69	49	13,415	8216	0.86	0.59	1.27
March 17–March 22	113	74	13,302	8142	0.93	0.69	1.27
March 28–April 15	106	48	13,196	8094	1.35	0.95	1.95
April 6–April 16	97	60	13,099	8034	0.99	0.71	1.39
April 17–April 27	127	50	12,972	7984	1.56	1.12	2.22
April 28–May 7	81	59	12,891	7925	0.84	0.6	1.2
May 8–May 22	135	76	12,756	7849	1.09	0.82	1.47
**>80**							
From pandemic inception to March 16	56	24	11,021	1057	0.22	0.14	0.38
March 17–March 22	118	68	10,903	989	0.16	0.11	0.22
March 28–April 15	139	64	10,764	925	0.19	0.14	0.26
April 6–April 16	149	46	10,615	879	0.27	0.19	0.38
April 17–April 27	130	48	10,485	831	0.21	0.15	0.31
April 28–May 7	87	40	10,398	791	0.17	0.11	0.25
May 8–May 22	125	28	10,273	763	0.33	0.22	0.52

OR: Odds ratios representing vaccinated vs not vaccinate likelihood of being tested for SARS-CoV-2 at each time interval.

For Males : Pooled odds ratio = 1.02 (95% CI = 0.96 to 1.08)

I_2 (inconsistency) = 96.7% (95% CI = 96.2% to 97.1%)

Females' probability of undergoing SARS-CoV-2 swab test for vaccinated vs unvaccinated, by age group and time from pandemic onset to May 22

OR: Odds ratios representing vaccinated vs unvaccinated likelihood of being tested for SARS-CoV-2 at each time interval

Stratum	*N* Vaccinated Tested	*N* Unvaccinated Tested	*N* Total Vaccinated	*N* Total Unvaccinated	OR	L95%CI	U95%CI
**<50**							
From pandemic inception to March 16	34	183	5117	143,940	5.23	3.51	7.58
March 17–March 22	44	319	5073	143,621	3.9	2.78	5.37
March 28–April 15	38	451	5035	143,170	2.4	1.67	3.34
April 6–April 16	68	783	4967	142,387	2.49	1.91	3.2
April 17–April 27	59	801	4908	141,586	2.12	1.6	2.77
April 28–May 7	44	576	4864	141,010	2.21	1.59	3.02
May 8–May 22	53	677	4811	140,333	2.28	1.69	3.03
**51–60**							
From pandemic inception to March 16 female	24	87	5108	38,750	2.09	1.27	3.32
March 17–March 22	41	151	5067	38,599	2.07	1.43	2.94
March 28–April 15	35	167	5032	38,432	1.6	1.08	2.32
April 6–April 16	42	277	4990	38,155	1.16	0.82	1.61
April 17–April 27	53	267	4937	37,888	1.52	1.11	2.06
April 28–May 7	31	147	4906	37,741	1.62	1.06	2.41
May 8–May 22	41	229	4865	37,512	1.38	0.96	1.93
**61–70**							
From pandemic inception to March 16	30	47	9838	21,596	1.4	0.86	2.26
March 17–March 22	41	73	9797	21,523	1.23	0.82	1.83
March 28–April 15	45	72	9752	21,451	1.37	0.92	2.02
April 6–April 16	69	59	9683	21,392	2.58	1.8	3.72
April 17–April 27	87	113	9596	21,279	1.71	1.27	2.28
April 28–May 7	61	109	9535	21,170	1.24	0.89	1.72

May 8–May 22	70	70	9465	21,100	2.23	1.58	3.15
**71–80**							
From pandemic inception to March 16	33	25	14,389	10,967	1.01	0.58	1.77
March 17–March 22	74	59	14,315	10,908	0.96	0.67	1.37
March 28–April 15	72	59	14,243	10,849	0.93	0.65	1.34
April 6–April 16	96	61	14,147	10,788	1.2	0.86	1.68
April 17–April 27	103	67	14,044	10,721	1.17	0.85	1.62
April 28–May 7	80	45	13,964	10,676	1.36	0.93	2.01
May 8–May 22	111	81	13,853	10,595	1.05	0.78	1.42
**>80**							
From pandemic inception to March 16	42	22	17,756	2921	0.31	0.18	0.55
March 17–March 22	147	59	17,609	2862	0.4	0.3	0.56
March 28–April 15	362	141	17,247	2721	0.41	0.33	0.5
April 6–April 16	282	127	16,965	2594	0.34	0.27	0.42
April 17–April 27	279	131	16,686	2463	0.31	0.25	0.39
April 28–May 7	135	59	16,551	2404	0.33	0.24	0.46
May 8–May 22	125	76	16,426	2328	0.23	0.17	0.32

For Females; Pooled odds ratio = 1.06 (95% CI = 1 to 1.12)
I_2 (inconsistency) = 96.4% (95% CI = 95.8% to 96.8%)

Section S2. Characteristics of individuals undergoing SARS-CoV-2 swab test from pandemic inception to May 22, 2020 in the Italian province of Reggio Emilia.

		Timing of SARS-CoV-2 Test													
		From Outbreak Onset to March 16		March 17–March 27		March 28–April 5		April 6–April 16		April 17–April 27		April 28–May 7		May 8– May 22	
		N	%	*N*	%	*N*	%	*N*	%	*N*	%	*N*	%	*N*	%
**Sex**	female	534	47.3	1024	49.1	1453	60.8	1929	59.8	1967	57.2	1254	52.7	1614	51.9
	male	595	52.7	1063	50.9	936	39.2	1297	40.2	1472	42.8	1126	47.3	1496	48.1
Age	<50	383	33.9	662	31.7	815	34.1	1451	45.0	1557	45.3	1153	48.4	1407	45.2
	50-60	235	20.8	402	19.3	349	14.6	507	15.7	563	16.4	350	14.7	511	16.4
	61-70	188	16.7	305	14.6	230	9.6	343	10.6	379	11.0	286	12.0	425	13.7
	71-80	178	15.8	324	15.5	287	12.0	317	9.8	350	10.2	267	11.2	411	13.2
	>80	145	12.8	394	18.9	708	29.6	608	18.8	590	17.2	324	13.6	356	11.4
Charlson Index	0	676	59.9	1298	62.2	1366	57.2	2139	66.3	2275	66.2	1584	66.6	1979	63.6
	1	83	7.4	189	9.1	242	10.1	245	7.6	258	7.5	139	5.8	220	7.1
	2	76	6.7	169	8.1	211	8.8	244	7.6	246	7.2	166	7.0	205	6.6
	≥3	119	10.5	203	9.7	288	12.1	282	8.7	284	8.3	188	7.9	235	7.6
	Unknown	175	15.5	228	10.9	282	11.8	316	9.8	376	10.9	303	12.7	471	15.1
Exposed to influenza vaccination	No	751	66.5	1351	64.7	1447	60.6	2254	69.9	2423	70.5	1718	72.2	2211	71.1
	Yes	366	32.4	713	34.2	918	38.4	953	29.5	994	28.9	630	26.5	849	27.3
	Unknown	12	1.1	23	1.1	24	1.0	19	.6	22	0.6	32	1.3	50	1.6
Type of vaccine	Trivalent	93	8.2	209	10.0	452	18.9	374	11.6	312	9.1	184	7.7	222	7.1
	Tetravalent	247	21.9	464	22.2	425	17.8	527	16.3	608	17.7	414	17.4	583	18.7
Time of exposure to vaccine	October 2019	56	5.0	112	5.4	119	5.0	166	5.1	151	4.4	104	4.4	131	4.2
	November 2019	264	23.4	515	24.7	662	27.7	623	19.3	708	20.6	435	18.3	599	19.3
	December 2019–	46	4.1	86	4.1	137	5.7	164	5.1	135	3.9	91	3.8	119	3.8

	March 2020														
Prevalence positive		687	60.9	1433	68.7	1175	49.2	916	28.4	471	13.7	123	5.2	91	2.9

Section S3. Time from diagnosis to hospital admission (Figure S1) and to death (Figure S2, next page) for covid-19 patients exposed and unexposed to influenza vaccination.

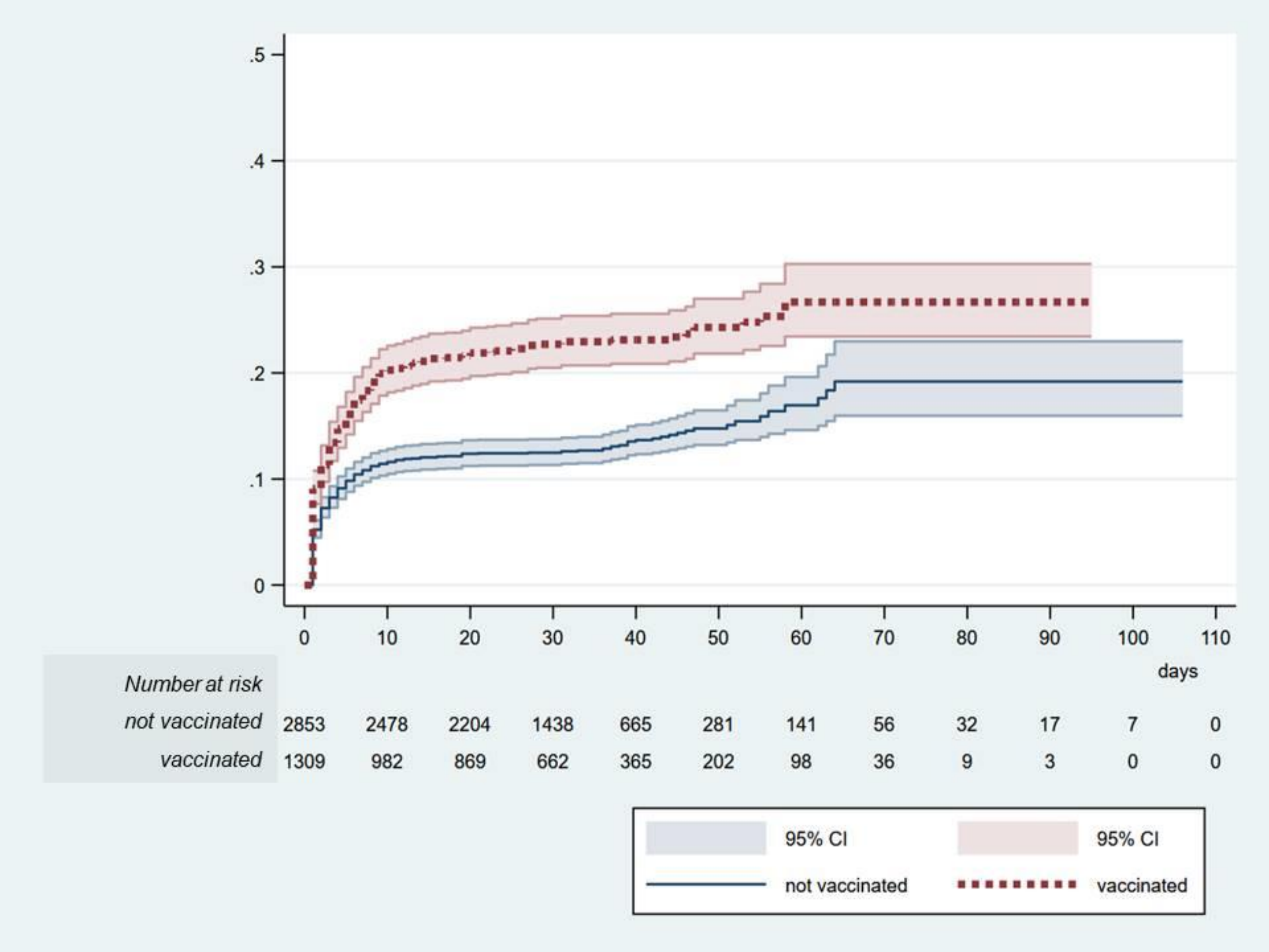

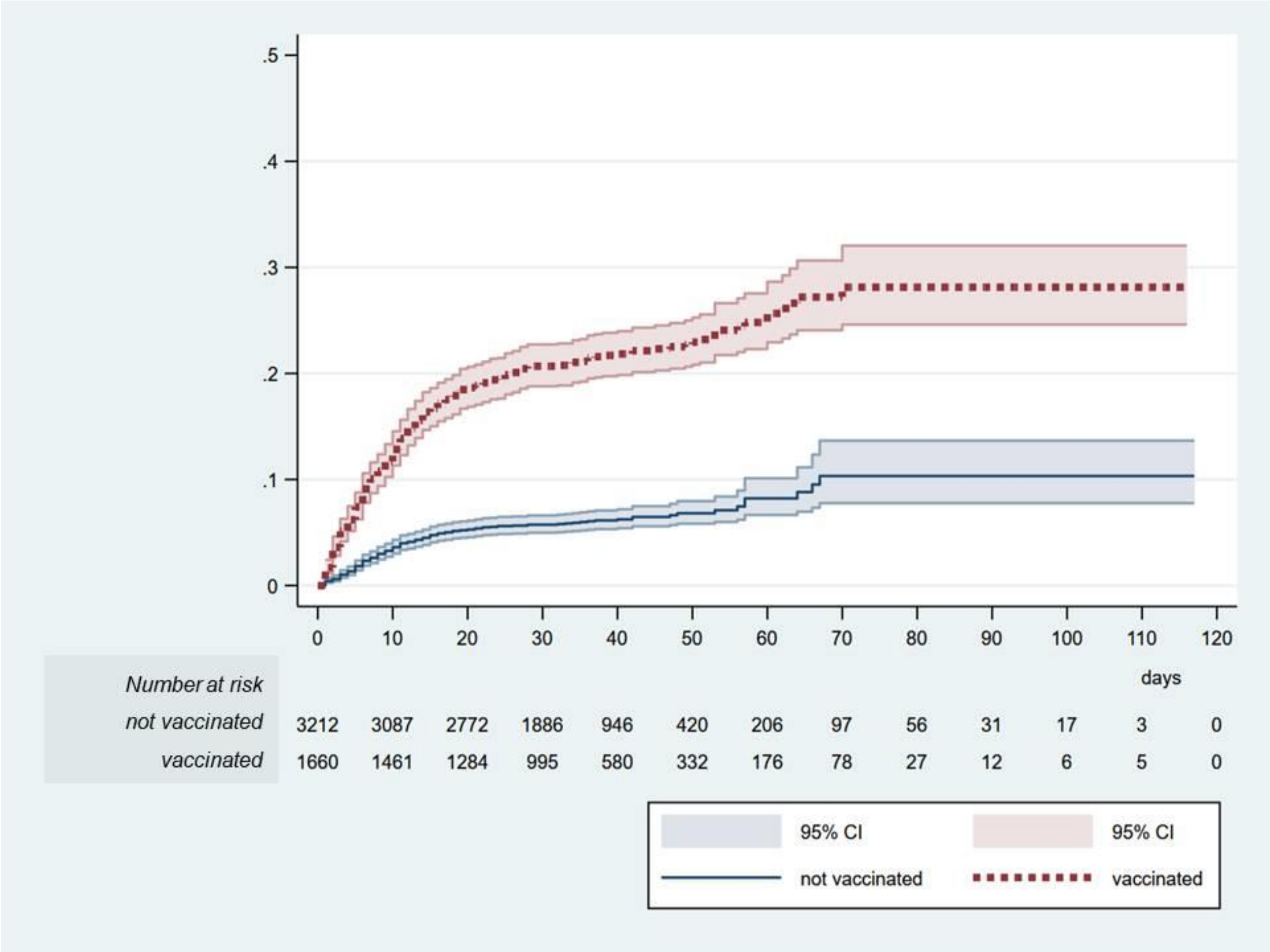

.5
.4
.3
.2
.1
0
0 10 20 30 40 50 60 70 80 90 100 110 120
days
Number at risk
not vaccinated 3212 3087 2772 1886 946 420 206 97 56 31 17 3 0
vaccinated 1660 1461 1284 995 580 332 176 78 27 12 6 5 0
95% CI
not vaccinated
95% CI
vaccinated
